# A Reliability Test of a Complex System Based on Empirical Likelihood

**DOI:** 10.1371/journal.pone.0163557

**Published:** 2016-10-19

**Authors:** Yan Zhou, Liya Fu, Jun Zhang, Yongchang Hui

**Affiliations:** 1 College of Mathematics and Statistics, Institute of Statistical Sciences, Shenzhen University, Shenzhen, China; 2 School of Mathematics and Statistics, Xi’an Jiaotong University, Xi’an, China; 3 College of Mathematics and Computational Science, Institute of Statistical Sciences, Shen Zhen-Hong Kong Joint Research Center for Applied Statistical Sciences, Shenzhen University, Shenzhen, China; State University of New York, UNITED STATES

## Abstract

To analyze the reliability of a complex system described by minimal paths, an empirical likelihood method is proposed to solve the reliability test problem when the subsystem distributions are unknown. Furthermore, we provide a reliability test statistic of the complex system and extract the limit distribution of the test statistic. Therefore, we can obtain the confidence interval for reliability and make statistical inferences. The simulation studies also demonstrate the theorem results.

## Introduction

In practice, little information can be directly derived from a complex system (CS). What we usually obtain is information about subsystems. Therefore, system reliability, which is based on subsystem data, is a very important research topic and has been a concern for a long time. However, due to the complexity of the system, life distribution, subsystem reliability, and diversity of the data distribution, there are many difficulties studying of a CS.

In recent years, many researchers have provided various methods for calculating and estimating system reliability under the assumption that the distribution of the subsystems (family) is known. For example, for a series system with two subsystems and with binomially distributed pass-fail failure data, Buehler [1] proposed a model and derived the exact lower confidence limit of the system reliability. Rosenblatt [2] proposed an approximate method for system reliability confidence limits based on the asymptotic normality of a U-statistic. Weaver [3] derived a simple and accurate ordering method to calculate the system reliability confidence limits, but it requires the same sample size for all subsystems. Rice and Moore [4] presented a Monte Carlo model for binomial distributed data that is valid for a zero failure case. Coit [5] provided a method which does not require any parametric assumptions for component reliability or time to failure. Tian [6] summarized and compared the advantages and limitations of the parametric methods.

However, only a few studies have investigated the reliability test of a CS. The main reasons are that the life distributions of the subsystems may vary, and it is difficult to find out which one it is. It is not easy to find a test statistic and its asymptotic distribution because of the complexity of the system structures.

In recent studies, regarding the series system in which the subsystems’ life distribution is an index distribution, Yu et al. [7] considered the system failure rate of testing and gave an accurate unbiased test. Based on the method proposed by Yu et al. [7], Li [8] further considered subsystem lives following different distributions for the series system and completed the corresponding test by approximately transforming the non-index distribution to index distribution.

In a normal situation, it is difficult to determine the life distribution of a subsystem, and the structure of the system may be very complicated. In this paper, we assume the following: (i) A complex system is described by minimal paths, and these minimal paths are known; (ii) The subsystems of the complex system are independent, and the distribution of life is unknown. Under these two assumptions, we provide a reliability test statistic for a complex system using the empirical likelihood method [9, 10]. Furthermore, we also extract the limit distribution of the test statistic. Therefore, we can obtain the confidence interval and make statistical inferences for the system reliability based on the limit distribution.

This paper is organized as follows. In Section 2, we describe the reliability test problem of the complex system. In Section 3, we provide a test statistic and derive its asymptotic distribution. In Section 4, we carry out simulation studies for a bridge system. Finally, we draw several conclusions. The proof of the asymptotic distribution is given in the Appendix.

## Materials and Methods

### Notations

The life of a complex system is *Z* = max_1≤*j*≤*k*_ min_*r*∈Ω_*j*__ *Z*_*r*_, where *k* is the number of minimal paths, Ω_*j*_ is the *jth* minimal path, and *Z*_*r*_ is the life of the *r*th subsystem. The specific expression of complex system reliability has been derived in [11, 12]. For convenience, we first define some operations and give some notions.

**Definition 2.1**: Let *α* = (*a*_1_, *a*_2_, …, *a*_*m*_)^*T*^ and *β* = (*b*_1_, *b*_2_, …, *b*_*m*_)^*T*^ be two column vectors with *m* elements.

Define *α* ≺ *β* if *a*_*i*_ ≤ *b*_*i*_, *i* = 1, 2, …, *m*;

Define *α* \ *β* = (*δ*_1_, *δ*_2_, …, *δ*_*m*_)^*T*^, where $\delta_{i}=\{\begin{array}{cc} 1 & \;\;\;a_{i}=1,b_{i}=0\\ 0 & \;\;\;\text{others} \end{array}$, *i* = 1, 2, …, *m*;

Define *α* ⊕ *β* = (*γ*_1_, *γ*_2_, …, *γ*_*m*_)^*T*^, where *γ*_1_ = *a*_1_ + *b*_1_ and *γ*_*i*_ = max{*a*_*i*_, *b*_*i*_} for *i* = 2, …, *m*.

**Definition 2.2**: Let *A* = (*α*_1_, *α*_2_, …, *α*_*k*_) be an *m* × *k* matrix, and *A*_*j*_ = (*α*_1_ \ *α*_*j*_, *α*_2_ \ *α*_*j*_, …, *α*_*j*−1_ \ *α*_*j*_) for a given *j*, where *j* = 2, …, *k*. If *α*_*t*_1__ \ *α*_*j*_ ≺ *α*_*t*_ \ *α*_*j*_ for all *t*_1_ < *t* ≤ *j*, then we cut out the column *α*_*t*_ \ *α*_*j*_ of *A*_*j*_. Denote the gained matrix by $A_{j}^{*}$, which is an *m* × *n*_*j*_ matrix, where *n*_*j*_ ≤ *j* − 1 and *j* = 2, 3, …, *k*. Define $V\left(A_{j}^{*}\right)=\max_{1\leq s\leq m}\sum_{t=1}^{n_{j}}A_{j}^{*}\left(s,t\right)$, where $A_{j}^{*}\left(s,t\right)$ is the *s* row and the *t* column element of $A_{j}^{*}$.

**Definition 2.3**: Let *C* = (*c*_1_, *c*_2_, …, *c*_*k*_) be an *m* × *k* matrix. For a fixed index *j*, *j* = 1, 2, …, *k*, define *f*_*i*_1_*i*_2_,…,*i*_*j*__ = *c*_*i*_1__ ⊕ *c*_*i*_2__ ⊕ … ⊕ *c*_*i*_*j*__, where 1 ≤ *i*_1_ < *i*_2_ < … < *i*_*j*_ ≤ *k*. Let ${\tilde{C}}_{j}=\left(f_{i_{1}i_{2}\ldots i_{j}}\right)$ be an $m\times C_{k}^{j}$ matrix whose column vectors are made of all vectors *f*_*i*_1_*i*_2_…*i*_*j*__, where $C_{k}^{j}$ is the combination number of *j* in *k*.

**Definition 2.4**: Let *α* = (*a*_1_, *a*_2_, …, *a*_*m*_)^*T*^ and *R* = (*r*_1_, *r*_2_, …, *r*_*m*_)^*T*^ be vectors with *m* elements, and *D* = (*d*_1_, *d*_2_, …, *d*_*k*_) be an *m* × *k* matrix. Define

$$\begin{array}{c} R^{\alpha }=\prod_{i=1}^{m}r_{i}^{a_{i}}\;\;\text{and}\;\;R^{D}=\sum_{j=1}^{k}R^{d_{j}}. \end{array}$$

Based on these definitions, the reliability function for a system can be calculated with the following theorem [11].

**Theorem 2.5**: Let *F*_*r*_(*t*) be a life distribution function for an independent subsystem *S*_*r*_, and *R*_*r*_(*t*) = 1 − *F*_*r*_(*t*) is the reliability of the *r*th subsystem, *r* = 1, 2, …, *m*. The minimal path matrix of a complex system is *A*_*m*×*k*_, and *F*_1,*A*_(*t*) is the life function for the complex system. Ψ_1,*A*_(*t*) = 1 − *F*_1,*A*_(*t*) is the reliability function. Define

$$\begin{array}{c} C=(\begin{array}{c} 1^{T}\\ A \end{array})=\left(c_{1},c_{2},\ldots ,c_{k}\right), \end{array}$$

where **1** is a vector of 1s, Thus,

$$\begin{array}{c} \Psi_{1,A}\left(t\right)=-\sum_{j=1}^{k}\tilde{R}{\left(t\right)}^{{\tilde{C}}_{j}}, \end{array}$$

where $\tilde{R}\left(t\right)={\left\{-1,R_{1}\left(t\right),R_{2}\left(t\right),\ldots ,R_{m}\left(t\right)\right\}}^{T}$.

In view of the characteristics of the minimal path matrix, the calculation procedure can be cut short; Thus, we can obtain the following corollary.

**Corollary**: According to Zhang et al. [11], suppose that there are *r*_*i*_ zeroes in the *i*th row of *A* in Theorem 2.5, where *i* = 1, …, *m*. Let *l* = *max*_1 ≤ *i* ≤ *m*_ *r*_*i*_. Then

$$\begin{array}{c} \Psi_{1,A}\left(t\right)=-\sum_{j=1}^{l}{\tilde{R}}^{{\tilde{C}}_{j}}\left(t\right)-\sum_{j=l+1}^{k}{\left(-1\right)}^{j}C_{k}^{j}\prod_{i=1}^{m}R_{i}\left(t\right). \end{array}$$

## A complex system reliability test based on the empirical likelihood

To infer the reliability lower confidence limit or the confidence limit of a CS using subsystem data, we can construct the following hypothesis tests: For a given *t*, test whether Ψ_1,*A*_(*t*) is not less than Ψ_0_ or not; that is,

$$\begin{array}{c} \left(1\right)\;\;H_{0}:\Psi_{1,A}\left(t\right)=\Psi_{0}\;\text{vs}\;H_{1}:\Psi_{1,A}\left(t\right)\neq \Psi_{0}; \end{array}$$

$$\begin{array}{c} \left(2\right)\;\;H_{0}:\Psi_{1,A}\left(t\right)\geq \Psi_{0}\;\text{vs}\;H_{1}:\Psi_{1,A}\left(t\right)<\Psi_{0}. \end{array}$$

We use empirical likelihood (EL), which is a nonparametric method introduced by Owen [9, 10, 13], to test the two hypotheses. Here, we first give a short introduction to empirical likelihood. The definitions of the empirical distribution function and the empirical accumulate function are given as follows.

**Definition 3.1**: Let $X_{1},X_{2},\ldots ,X_{n}\in R$ be independently identically distributed, then the empirical cumulative distribution function of *X*_1_, *X*_2_, …, *X*_*n*_ is

$$\begin{array}{c} F_{n}\left(x\right)=\frac{1}{n}\sum_{i=1}^{n}I_{\left(X_{i}\leq x\right)}, \end{array}$$

for −∞ < *x* < ∞.

**Definition 3.2**: Let $X_{1},\;X_{2},\ldots ,X_{n}\in R$, be independent and with a common cumulative distribution *F*, the nonparametric likelihood of the *F* is

$$\begin{array}{c} L\left(F\right)=\Pi_{i=1}^{n}\left(F\left(X_{i}\right)-F\left(X_{i}-\right)\right). \end{array}$$

Define

$$\begin{array}{c} R\left(F\right)=\frac{L(F)}{L(F_{n})}. \end{array}$$

Then the empirical likelihood ratio statistic is defined to be

$$\begin{array}{c} ℜ\left(\mu_{0}\right)=\max\{\Pi_{i=1}^{n}nw_{i}|\sum_{i=1}^{n}w_{i}\left(X_{i}-\mu_{0}\right)=0,w_{i}\geq 0,\sum_{i=1}^{n}w_{i}=1\}. \end{array}$$

The resulting empirical likelihood confidence region for the mean *μ*_0_ is

$$\begin{array}{c} \left\{\mu |R\left(\mu_{0}\right)\geq r_{0}\right\}=\arg\max\{\sum_{i=1}^{n}w_{i}X_{i}|\Pi_{i=1}^{n}nw_{i}\geq r_{0},w_{i}\geq 0,\sum_{i=1}^{n}w_{i}=1\}. \end{array}$$

For a given hypothesis test, we can obtain a confidence region with the empirical likelihood method without assuming the specification of the data distribution family. In the following two subsections, we will derive the test statistics for two hypothesis tests for the reliability of a complex system using the empirical likelihood method.

### Two-sided hypothesis test for the reliability of a CS

In this subsection, we consider a two-sided hypothesis test for the reliability of a complex system. For a given *t*,

$$\begin{array}{c} H_{0}:\Psi_{1,A}\left(t\right)=\Psi_{0}\;\;\;\text{vs}\;\;\;H_{1}:\Psi_{1,A}\left(t\right)\neq \Psi_{0}. \end{array}$$

According to Theorem 2.5, we have $\Psi_{1,A}\left(t\right)=-\sum_{j=1}^{k}\sum_{l=1}^{C_{k}^{j}}{\left(-1\right)}^{j}\Pi_{r=1}^{m}{\left[R_{r}\left(t\right)\right]}^{{\tilde{c}}_{r,l}^{j}},$ where ${\tilde{c}}_{r,l}^{j}$ equals 0 or 1, and Ψ_1,*A*_(*t*) is a smoothing function of *R*_1_(*t*), …, *R*_*m*_(*t*). We know that *R*_1_(*t*), …, *R*_*m*_(*t*) are independent of each other for the independent subsystems, and use *I*_(*Z*_*ir*_ > *t*)_ to replace *R*_*r*_(*t*), where *Z*_*ir*_ is the observed lifetime of the *r*th subsystem of the *i*th sample. For any *t* > 0, we have

$$\begin{array}{ccc} E\Psi_{1,A}\left(Z_{i},t\right) & = & -E\{\sum_{j=1}^{k}\sum_{l=1}^{C_{k}^{j}}{\left(-1\right)}^{j}\Pi_{r=1}^{m}{\left[{\hat{R}}_{r}\left(t\right)\right]}^{{\tilde{c}}_{r,l}^{j}}\}\\ & = & -\sum_{j=1}^{k}\sum_{l=1}^{C_{k}^{j}}{\left(-1\right)}^{j}\Pi_{r=1}^{m}E\{{\left[{\hat{R}}_{r}\left(t\right)\right]}^{{\tilde{c}}_{r,l}^{j}}\}\\ & = & -\sum_{j=1}^{k}\sum_{l=1}^{C_{k}^{j}}{\left(-1\right)}^{j}\Pi_{r=1}^{m}E\left\{{\left[I_{\left(Z_{ir}>t\right)}\right]}^{{\tilde{c}}_{r,l}^{j}}\right\}\\ & = & -\sum_{j=1}^{k}\sum_{l=1}^{C_{k}^{j}}{\left(-1\right)}^{j}\Pi_{r=1}^{m}{\left[R_{r}\left(t\right)\right]}^{{\tilde{c}}_{r,l}^{j}}\\ & = & \Psi_{1,A}\left(t\right). \end{array}$$

Under the null hypothesis, we obtain

$$\begin{array}{c} \sum_{i=1}^{n}w_{i}\left[\Psi_{1,A}\left(Z_{i},t\right)-\Psi_{0}\right]=0. \end{array}$$

Therefore, a statistic for the two-sided test based on the EL is given as follows:

$$\begin{array}{c} ℜ\left(\Psi_{0}\right)=\max\{\prod_{i=1}^{n}nw_{i}|\sum_{i=1}^{n}w_{i}\left[\Psi_{1,A}\left(Z_{i},t\right)-\Psi_{0}\right]=0,\;w_{i}\geq 0,\sum_{i=1}^{n}w_{i}=1\}. \end{array}$$

Let

$$\begin{array}{c} G=\sum_{i=1}^{n}\log nw_{i}-n\lambda^{T}\sum_{i=1}^{n}w_{i}\left[\Psi_{1,A}\left(Z_{i},t\right)-\Psi_{0}\right]+\mu \left(\sum_{i=1}^{n}w_{i}-1\right), \end{array}$$

where *λ* and *μ* are Lagrange multipliers. The estimating function based on the derivative of *G* with respect to *w*_*i*_ is

$$\begin{array}{c} \frac{1}{w_{i}}-n\lambda^{T}\left(\Psi_{1,A}\left(Z_{i},t\right)-\Psi_{0}\right)+\mu =0. \end{array}$$

Because $\sum_{i=1}^{n}w_{i}\frac{\partial G}{\partial w_{i}}=0$, then *μ* = −*n*, we have

$$\begin{array}{c} w_{i}=\frac{1}{n}\frac{1}{1+\lambda [\Psi_{1,A}\left(Z_{i},t\right)-\Psi_{0}]}. \end{array}$$

To solve *λ* from the following equation

$$\begin{array}{c} \sum_{i=1}^{n}\frac{1}{n}\frac{\Psi_{1,A}\left(Z_{i},t\right)-\Psi_{0}}{1+\lambda [\Psi_{1,A}\left(Z_{i},t\right)-\Psi_{0}]}=0, \end{array}$$

let $n_{1}=\sum_{i=1}^{n}\Psi_{1,A}\left(Z_{i},t\right)$, then

$$\begin{array}{c} \frac{n_{1}\left(1-\Psi_{0}\right)}{1+\lambda (1-\Psi_{0})}-\frac{\left(n-n_{1}\right)\Psi_{0}}{1-\lambda \Psi_{0}}=0. \end{array}$$

From the above equation, we can obtain

$$\begin{array}{c} \lambda =\frac{1}{n}\frac{n_{1}-n\Psi_{0}}{\Psi_{0}\left(1-\Psi_{0}\right)}. \end{array}$$

Therefore, we can derive

$$\begin{array}{c} -2\log ℜ\left(\Psi_{0}\right)=n_{1}\log\frac{n_{1}}{n\Psi_{0}}+\left(n-n_{1}\right)\log\frac{\left(n-n_{1}\right)}{n(1-\Psi_{0})}. \end{array}$$

Then, we can obtain the following theorem from the EL method.

**Theorem 3.1**: Random variables *Z*_1_, *Z*_2_, …, *Z*_*n*_ are independent random vectors of the *m*-dimension with a common distribution *F*_0_. $\Psi_{1,A}\left(Z_{i},t\right)\in R^{1},$ and $\Psi_{0}\in R^{1}$. Let $m\left(Z,\Psi_{0}\right)=\Psi_{1,A}\left(Z,t\right)-\Psi_{0}\in R^{1}$. Under the null hypothesis, *E*(*m*(*Z*, Ψ_0_)) = 0 and *var*(*m*(*Z*, *ψ*_0_)) < 1. Then −2logℜ(Ψ_0_) converges to $\chi_{1}^{2}$ in distribution as *n* → ∞.

The proof of Theorem 3.1 is similar to Owen [10]. Let $\chi_{(1).95}^{2}$ be the 95% quantile of the chi-square distribution with one degree of freedom. According to Theorem 3.1, we have

$$\begin{array}{c} \lim_{n\to \infty }p\left\{-2\log ℜ\left(\Psi_{0}\right)>\chi_{(1).95}\right\}=0.05. \end{array}$$

### One-sided hypothesis test for the reliability of a CS

For the reliability of a complex system, we are more interested in whether the reliability of the system is no less than a given value, that is, for a given *t*,

$$\begin{array}{c} H_{0}:\Psi_{1,A}\left(t\right)\geq \Psi_{0}\;\;\;\;\;vs\;\;\;\;\;H_{1}:\Psi_{1,A}\left(t\right)<\Psi_{0}. \end{array}$$

Under the same condition as Theorem 3.1, we can obtain the following theorem.

**Theorem 3.2**: Let *Z*_1_, *Z*_2_, …, *Z*_*n*_ be independent random vectors with a common and unknown distribution *F*_0_, and $Z_{i}\in R^{m}$ for *i* = 1, …, *n*. Let *I*(*Z*_*i*_ < *t*) = {*I*(*Z*_1*i*_ < *t*), …, *I*(*Z*_*mi*_ < *t*)}^*T*^ and *EI*(*Z*_*i*_ < *t*) = 1 − *R*(*t*), where *R*(*t*) = {*R*_1_(*t*), …, *R*_*m*_(*t*)}^*T*^. Let Ψ_1,*A*_(*Z*, *t*) be a function that maps $R^{m}$ to $R^{1}$. Let *E*(Ψ_1,*A*_(*Z*, *t*)) = Ψ_1_ and *var*(Ψ_1,*A*_(*Z*, *t*)) < ∞. Then under the null hypothesis, for any *t* > 0,

$$\begin{array}{c} \lim_{n\to \infty }p\left\{-2\log ℜ\left(\Psi_{0}\right)\leq c\right\}=\{\begin{array}{cc} \frac{1}{2}+\frac{1}{2}p\left\{\chi_{1}^{2}\leq c\right\} & \;\;\;\Psi_{1,A}\left(t\right)=\Psi_{0}\\ 1 & \;\;\;\Psi_{1,A}\left(t\right)>\Psi_{0}, \end{array} \end{array}$$

where *c* is a constant.

The proof of Theorem 3.2 is given in the Appendix. According to Theorem 3.2, if we take 0.05 as a remarkable level, first, we should make a judgment of the Ψ_1,*A*_(*X*, *t*) average value according to the certificate process: If $\sum_{i=1}^{n}\Psi_{1,A}\left(Z_{i},t\right)/n>\Psi_{0}$, then we should accept *H*_0_; otherwise, we should take $c=\chi_{(1).95}^{2}$ as a threshold value and then check whether −2logℜ(Ψ_0_) is larger than *c*. If −2logℜ(Ψ_0_) < *c*, then accept the null hypothesis; otherwise, we will reject the null hypothesis.

## Simulation studies

**Fig 1:**
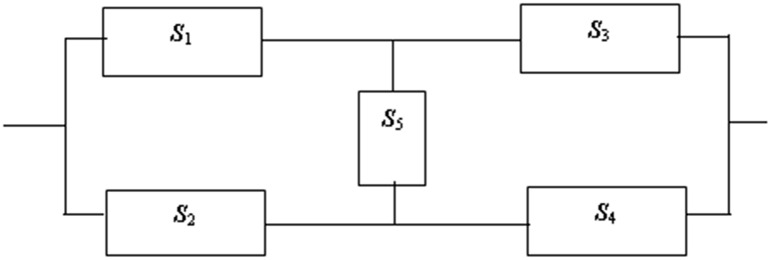
The branching program of a bridge-type complex system.

In this section, we carry out simulation studies to assess the performance of the proposed method. We take *α* = 0.05 and *α* = 0.1 as remarkable levels, compare the power under the alternative hypothesis under controlling type I error, and check whether the result is close to the theory result as the sample size increases. We consider ten different sample sizes, specifically, *n* = 10, 15, 20, 30, 50, 60, 80, 100, 150, and 300. For each case, 10000 replications are carried out. We generate samples of each subsystem from $\chi_{20}^{2}$ and take *t* = 15 as an example. This test is not related to the system’s degree of complexity, and we select a simple bridge-type complex system for the simulation studies. The expression of the bridge-type complex system used by Zhang et al. [11] is given in Fig 1.

There are four minimal paths in the above system: {*s*_1_, *s*_2_}, {*s*_3_, *s*_4_}, {*s*_1_, *s*_4_, *s*_5_}, {*s*_2_, *s*_3_, *s*_5_}. Then, we have

$$\begin{array}{c} A=(\begin{array}{cccc} 1 & 0 & 1 & 0\\ 1 & 0 & 0 & 1\\ 0 & 1 & 0 & 1\\ 0 & 1 & 1 & 0\\ 0 & 0 & 1 & 1 \end{array})=\left(\alpha_{1},\alpha_{2},\alpha_{3},\alpha_{4}\right) \end{array}$$

and

$$\begin{array}{c} C=(\begin{array}{c} 1^{T}\\ A \end{array})=\left(c_{1},c_{2},c_{3},c_{4}\right)=(\begin{array}{cccc} 1 & 1 & 1 & 1\\ 1 & 0 & 1 & 0\\ 1 & 0 & 0 & 1\\ 0 & 1 & 0 & 1\\ 0 & 1 & 1 & 0\\ 0 & 0 & 1 & 1 \end{array}). \end{array}$$

The maximum of the number of element zero is two in every row of *A*. It is easy to calculate

$$\begin{array}{c} \tilde{C}=(\begin{array}{cccccccccc} 1 & 1 & 1 & 1 & 2 & 2 & 2 & 2 & 2 & 2\\ 1 & 0 & 1 & 0 & 1 & 1 & 1 & 1 & 0 & 1\\ 1 & 0 & 0 & 1 & 1 & 1 & 1 & 0 & 1 & 1\\ 0 & 1 & 0 & 1 & 1 & 0 & 1 & 1 & 1 & 1\\ 0 & 1 & 1 & 0 & 1 & 1 & 0 & 1 & 1 & 1\\ 0 & 0 & 1 & 1 & 0 & 1 & 1 & 1 & 1 & 1 \end{array}) \end{array}$$

and

$$\begin{array}{c} \tilde{R}\left(t\right)={\left(-1,R_{1}\left(t\right),R_{2}\left(t\right),\ldots ,R_{5}\left(t\right)\right)}^{T}. \end{array}$$

According to the Corollary, the system reliability Ψ_1,*A*_(*t*) is given as follows:

$$\begin{array}{ccc} \Psi_{1,A}\left(t\right) & = & R_{1}\left(t\right)R_{2}\left(t\right)+R_{3}\left(t\right)R_{4}\left(t\right)+R_{1}\left(t\right)R_{4}\left(t\right)R_{5}\left(t\right)+R_{2}\left(t\right)R_{3}\left(t\right)R_{5}\left(t\right)\\ & & -R_{1}\left(t\right)R_{2}\left(t\right)R_{3}\left(t\right)R_{4}\left(t\right)-R_{1}\left(t\right)R_{2}\left(t\right)R_{3}\left(t\right)R_{5}\left(t\right)-R_{1}\left(t\right)R_{2}\left(t\right)R_{4}\left(t\right)R_{5}\left(t\right)\\ & & -R_{1}\left(t\right)R_{3}\left(t\right)R_{4}\left(t\right)R_{5}\left(t\right)-R_{2}\left(t\right)R_{3}\left(t\right)R_{4}\left(t\right)R_{5}\left(t\right)+2R_{1}\left(t\right)R_{2}\left(t\right)R_{3}\left(t\right)R_{4}\left(t\right)R_{5}\left(t\right). \end{array}$$

When *t* takes a value of 15, Ψ_1,*A*_(*t*) = 0.889. We consider the following two hypotheses.

**Simulation 1**: *H*_0_: Ψ_1,*A*_(*t*) = 0.889 *vs* *H*_1_: Ψ_1,*A*_(*t*) ≠ 0.889

**Fig 2:**
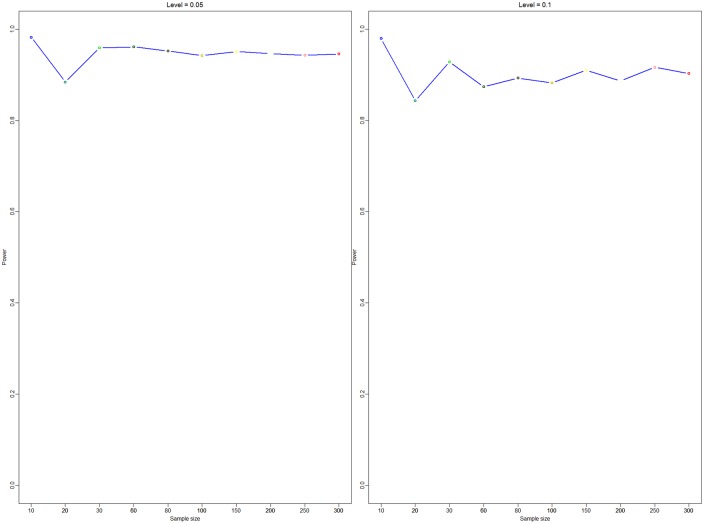
Test *H*_0_: Ψ_1,*A*_(*t*) = 0.889 *vs* *H*_1_: Ψ_1,*A*_(*t*) ≠ 0.889. The x-coordinate is the sample size, and the y-coordinate is power. The level of significance in the left panel is 0.05, and the level of significance in the right panel is 0.1.

Empirical likelihood does not depend on the distribution of samples but is related to Ψ_0_ which will be tested. The first simulation checks whether the type I error is controlled or not. Here, we take *n* = 10, 20, 30, 50, 80, 100, 50, 200, 250, and 300. It is easy to find that the accepted rate is well controlled about 0.95 when the level of significance is 0.05 (see the left panel in Fig 2), and the accepted rate is well controlled about 0.9 when the level of significance is 0.1 (see the right panel in Fig 2). The results are more accurate as the sample size increases.

**Simulation 2**: *H*_0_: Ψ_1,*A*_(*t*) ≥ Ψ_0_ *vs*. *H*_1_: Ψ_1,*A*_(*t*) < Ψ_0_

**Fig 3:**
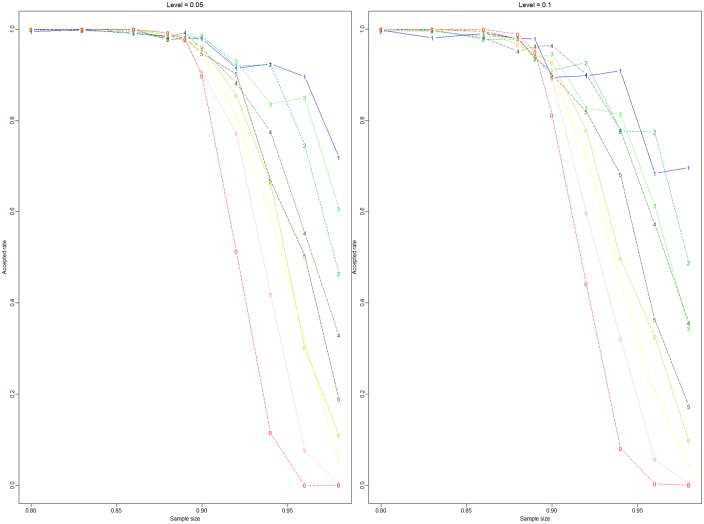
Test *H*_0_: Ψ_1,*A*_(*t*) ≥ Ψ_0_ *vs* *H*_1_: Ψ_1,*A*_(*t*) < Ψ_0_. Numbers 1, 2, 3, 4, 5, 6, 7, 8, 9, and 0 are across to the sample size 10, 15, 20, 30, 50, 60, 80, 100, 150, and 300, respectively. The x-coordinate is the value of Ψ_0_, and the y-coordinate is the accepted rate. The level of significance in the left panel is 0.05, and the level of significance in the right panel is 0.1.

**Fig 4:**
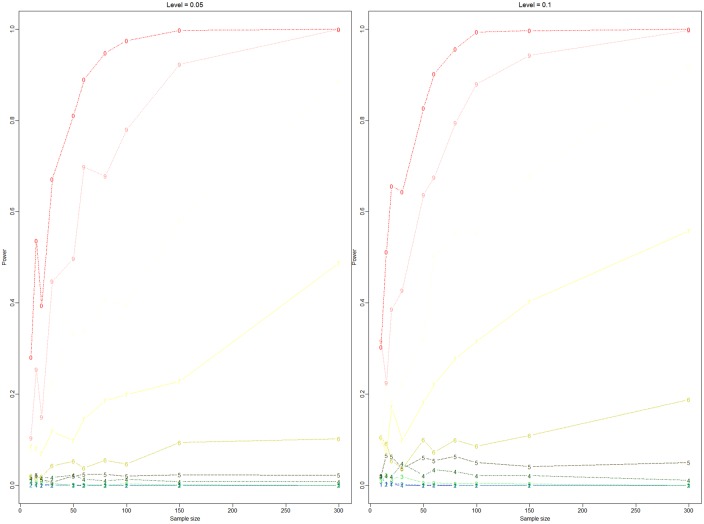
Test *H*_0_: Ψ_1,*A*_(*t*) ≥ Ψ_0_ *vs* *H*_1_: Ψ_1,*A*_(*t*) < Ψ_0_. Numbers 1, 2, 3, 4, 5, 6, 7, 8, 9, and 0 denote the powers of 0.80, 0.83, 0.86, 0.88, 0.89, 0.90, 0.92, 0.94, 0.96, and 0.98, respectively. The x-coordinate is the sample size, and the y-coordinate is power. The level of significance in the left panel is 0.05, and the level of significance in the right panel is 0.1.

The true value is 0.889 for the one-sided hypothesis test. In Fig 3, the sample size is fixed, and different values of Ψ_0_, 0.80, 0.83, 0.86, 0.88, 0.89, 0.90, 0.92, 0.94, 0.96, and 0.98, are considered. The sample size takes *n* = 10, 15, 20, 30, 50, 60, 80, 100, 150, and 300. Then, we compare the accepted rates of the different sample sizes in Fig 3. Fig 4 shows the power of the different sample sizes. Fig 3 shows that the accepted rate increases under the null hypothesis when the sample size increases and vice versa. Fig 4 shows that the rejected rate approaches 0 under the null hypothesis. The power increases when Ψ_0_ increases under the alternative hypothesis.

## Conclusion

This study on the reliability of a complex system described by minimal paths has theory significance, as well as practical application. However, there are few research results. Based on the life samples of subsystems, we use the empirical likelihood method to solve the reliability test problem, provide a reliability test statistic for a CS, and extract the limit distribution of the test statistic.

We carry out some simulation studies about a simple bridge-type complex system and obtain results. The simulation results are consistent with the theorem. Therefore, we use the EL to propose a statistic for the test of a complex system. This test statistic does not depend on the distribution of the samples, but is related to Ψ_0_, which will be tested, and is irrelevant to the degree of complexity of the system. In addition, it can control type I error well with a small sample size, and the power is consistent with the result when the sample size increases.

## Appendix

Proof of Theorem 3.2

Let *w* = (*w*_1_, *w*_2_, …, *w*_*n*_). It is easy to prove that function $f\left(w\right)=-2\log\left(\prod_{i=1}^{n}nw_{i}\right)$ is a convex function of *w*, and

$$\begin{array}{ccc} S_{1} & = & \{w|\sum_{i=1}^{n}w_{i}\left[\Psi_{1,A}\left(X_{i},t\right)-\Psi_{0}\right]=0,w_{i}\geq 0,\sum_{i=1}^{n}w_{i}=1\},\\ S_{2} & = & \left\{w|\sum_{i=1}^{n}w_{i}\left[\Psi_{1,A}\left(X_{i},t\right)-\Psi_{0}\right]\geq 0,w_{i}\geq 0,\sum_{i=1}^{n}w_{i}=1\right\} \end{array}$$

are closed convex sets; therefore there exist minimums in *S*_1_ and *S*_2_, respectively. Therefore, the minimums of *f*(*w*) exist in *S*_1_ and *S*_2_ by themselves. Let $g\left(w\right)=\sum_{i=1}^{n}w_{i}\left[\Psi_{1,A}\left(X_{i},t\right)-\Psi_{0}\right]$, where $\Psi_{1,A}\left(X_{i},t\right)=-\sum_{j=1}^{k}\sum_{l=1}^{C_{k}^{j}}{\left(-1\right)}^{j}\Pi_{r=1}^{m}E\left\{{\left[I_{\left(x_{r}>t\right)}\right]}^{{\tilde{c}}_{r,l}^{j}}\left(t\right)\right\}$. Let $h\left(w\right)=\sum_{i=1}^{n}w_{i}-1$ and $L\left(w\right)=\prod_{i=1}^{n}w_{i}$, we can obtain

$$\begin{array}{c} w^{\left(1\right)}=\arg_{w}\sup\left\{L\left(w\right)|\sum_{i=1}^{n}w_{i}\left[\Psi_{1,A}\left(X_{i},t\right)-\Psi_{0}\right]=0,w_{i}\geq 0,\sum_{i=1}^{n}w_{i}=1\right\}, \end{array}$$

$$\begin{array}{c} w^{\left(2\right)}=\arg_{w}\sup\left\{L\left(w\right)|\sum_{i=1}^{n}w_{i}\left[\Psi_{1,A}\left(X_{i},t\right)-\Psi_{0}\right]\geq 0,w_{i}\geq 0,\sum_{i=1}^{n}w_{i}=1\right\}. \end{array}$$

For the one-sided hypothesis test,

$$\begin{array}{c} H_{0}:\Psi_{1,A}\left(t\right)\geq \Psi_{0}\;\;\;\;\;\text{vs}\;\;\;\;\;H_{1}:\Psi_{1,A}\left(t\right)<\Psi_{0}, \end{array}$$

the nonparametric maximum likelihood ratio is

$$\begin{array}{c} ℜ\left(\Psi_{0}\right)=\frac{\sup\{L\left(w\right)|\sum_{i=1}^{n}w_{i}\left[\Psi_{1,A}\left(X_{i},t\right)-\Psi_{0}\right]\geq 0,w_{i}\geq 0,\sum_{i=1}^{n}w_{i}=1\}}{\sup\{L\left(w\right)|w_{i}\geq 0,\sum_{i=1}^{n}w_{i}=1\}}. \end{array}$$

The denominator is $\sup\left\{L\left(w\right)|w_{i}\geq 0,\;\sum_{i=1}^{n}w_{i}=1\right\}=\prod_{i=1}^{n}1/n,$ and

$$\begin{array}{c} L\left(w^{*}\right)=\sup\left\{L\left(w\right)|\sum_{i=1}^{n}w_{i}\left[\Psi_{1,A}\left(X_{i},t\right)-\Psi_{0}\right]\geq 0,\;w_{i}\geq 0,\;\sum_{i=1}^{n}w_{i}=1\right\}. \end{array}$$

The feasible region of problem (*MP*) is *D* = {*w*|*g*(*w*) ≥ 0}, also

$$\begin{array}{c} {\frac{\partial h(w)}{\partial w_{i}}|}_{w_{i}=w_{i}^{\left(2\right)}}=1,\;\;i=1,\ldots ,n, \end{array}$$

$$\begin{array}{c} {\frac{\partial g(w)}{\partial w_{i}}|}_{w_{i}=w_{i}^{\left(2\right)}}=\Psi_{1,A}\left(X_{i},t\right)-\Psi_{0}. \end{array}$$

Because vector (1, 1, …, 1)^*T*^ is independent of {[Ψ_1,*A*_(*X*_1_, *t*) − Ψ_0_], …, [Ψ_1,*A*_(*X*_*n*_, *t*) − Ψ_0_]}^*T*^, and functions *f*(*w*), *g*(*w*) and *h*(*w*) are first-order continuous differentiable and satisfy the Kuhn-Tucker theorem (necessary condition), then there exist λ* ≥ 0 and $\mu^{*}\in R^{1}$, which satisfy the following equations:

$$\begin{array}{c} \frac{\partial f}{\partial w_{i}}{|}_{w_{i}=w_{i}^{\left(2\right)}}-\lambda^{*}\frac{\partial g}{\partial w_{i}}{|}_{w_{i}=w_{i}^{\left(2\right)}}-\mu^{*}\frac{\partial h}{\partial w_{i}}{|}_{w_{i}=w_{i}^{\left(2\right)}}=0, \end{array}$$ (1)

$$\begin{array}{c} \lambda^{*}g\left(w^{\left(2\right)}\right)=0. \end{array}$$ (2)

From these equations, we can obtain μ* = −2*n*. Let *λ*^(2)^ = −λ*/2*n* ≤ 0, then

$$\begin{array}{c} w_{i}^{\left(2\right)}=\frac{1}{n}\frac{1}{1+\lambda^{\left(2\right)}\left[\Psi_{1,A}\left(X_{i},t\right)-\Psi_{0}\right]}. \end{array}$$ (3)

From Eq (2), we can derive

$$\begin{array}{c} \lambda^{\left(2\right)}g\left(w^{\left(2\right)}\right)=0. \end{array}$$ (4)

If *λ*^(2)^ = 0, we have $w_{i}^{\left(2\right)}=1/n$, and

$$\begin{array}{c} g\left(w^{\left(2\right)}\right)=\sum_{i=1}^{n}\frac{1}{n}\left[\Psi_{1,A}\left(X_{i},t\right)-\Psi_{0}\right]=\left(\bar{\Psi_{1,A}\left(X,t\right)}-\Psi_{0}\right)\geq 0. \end{array}$$ (5)

If *λ*^(2)^ < 0, we obtain from Eq (4)

$$\begin{array}{c} g(w^{\left(2\right)})=0, \end{array}$$ (6)

$$\begin{array}{ccc} & \Rightarrow & \sum_{i=1}^{n}\frac{1}{n}\frac{\Psi_{1,A}\left(X_{i},t\right)-\Psi_{0}}{1+\lambda^{\left(2\right)}\left[\Psi_{1,A}\left(X_{i},t\right)-\Psi_{0}\right]}=0,\\ & \Rightarrow & \sum_{i=1}^{n}\frac{1}{n}\left[\Psi_{1,A}\left(X_{i},t\right)-\Psi_{0}\right]-\sum_{i=1}^{n}\frac{1}{n}\frac{\lambda^{\left(2\right)}{\left[\Psi_{1,A}\left(X_{i},t\right)-\Psi_{0}\right]}^{2}}{1+\lambda^{\left(2\right)}\left[\Psi_{1,A}\left(X_{i},t\right)-\Psi_{0}\right]}=0,\\ & \Rightarrow & \lambda^{\left(2\right)}\sum_{i=1}^{n}\frac{1}{n}\frac{{\left[\Psi_{1,A}\left(X_{i},t\right)-\Psi_{0}\right]}^{2}}{1+\lambda^{\left(2\right)}\left[\Psi_{1,A}\left(X_{i},t\right)-\Psi_{0}\right]}=\left(\bar{\Psi_{1,A}\left(X,t\right)}-\Psi_{0}\right). \end{array}$$

Because *λ*^(2)^ < 0 and $w_{i}^{\left(2\right)}>0$, we have

$$\begin{array}{c} \lambda^{\left(2\right)}\sum_{i=1}^{n}w_{i}^{\left(2\right)}{\left[\Psi_{1,A}\left(X_{i},t\right)-\Psi_{0}\right]}^{2}<0\;\;\text{and}\;\;\bar{\Psi_{1,A}\left(X,t\right)}-\Psi_{0}<0, \end{array}$$

hence

$$\begin{array}{c} \lambda^{\left(2\right)}=0⟺\bar{\Psi_{1,A}\left(X,t\right)}-\Psi_{0}\geq 0. \end{array}$$ (7)

Due to $L\left(w^{\left(1\right)}\right)=\sup\left\{L\left(w\right)|\sum_{i=1}^{n}w_{i}\left[\Psi_{1,A}\left(X_{i},t\right)-\Psi_{0}\right]=0,\;w_{i}\geq 0,\;\sum_{i=1}^{n}w_{i}=1\right\}$, by the Lagrange multiplier

$$\begin{array}{c} w_{i}^{\left(1\right)}=\frac{1}{n}\frac{1}{1+\lambda^{\left(1\right)}\left[\Psi_{1,A}\left(X_{i},t\right)-\Psi_{0}\right]}, \end{array}$$

where *λ*^(1)^ satisfies the following equation,

$$\begin{array}{c} g\left(\lambda^{\left(1\right)}\right)=\sum_{i=1}^{n}\frac{1}{n}\frac{\Psi_{1,A}\left(X_{i},t\right)-\Psi_{0}}{1+\lambda^{\left(1\right)}\left[\Psi_{1,A}\left(X_{i},t\right)-\Psi_{0}\right]}=0. \end{array}$$

For *λ*^(2)^ < 0, we have

$$\begin{array}{c} w_{i}^{\left(2\right)}=\frac{1}{n}\frac{1}{1+\lambda^{\left(2\right)}\left[\Psi_{1,A}\left(X_{i},t\right)-\Psi_{0}\right]}\;\;\text{and}\;\;\;\;g\left(w^{\left(2\right)}\right)=0, \end{array}$$

hence

$$\begin{array}{c} g\left(\lambda^{\left(2\right)}\right)=\sum_{i=1}^{n}\frac{1}{n}\frac{\Psi_{1,A}\left(X_{i},t\right)-\Psi_{0}}{1+\lambda^{\left(2\right)}\left[\Psi_{1,A}\left(X_{i},t\right)-\Psi_{0}\right]}=0. \end{array}$$

Derivative with respect to *λ*, we have

$$\begin{array}{c} g^{\prime }\left(\lambda \right)=-\sum_{i=1}^{n}\frac{1}{n}\frac{{\left[\Psi_{1,A}\left(X_{i},t\right)-\Psi_{0}\right]}^{2}}{{\left\{1+\lambda \left[\Psi_{1,A}\left(X_{i},t\right)-\Psi_{0}\right]\right\}}^{2}}<0. \end{array}$$

Therefore, *g*(*λ*) is strictly monotone about *λ*; hence, when *λ*^(2)^ < 0 and *λ*^(2)^ = *λ*^(1)^, we have $w_{i}^{\left(2\right)}=w_{i}^{\left(1\right)}.$ Thus, $w_{i}^{\left(2\right)}=1/n$. When *λ*^(2)^ = 0, we have −2logℜ(Ψ_0_) = 0. According to Theorem 3.1, when *λ*^(2)^ < 0, −2logℜ(Ψ_0_) converges to $\chi_{1}^{2}$ in distribution. According to Eq (7), when $\bar{\Psi_{1,A}\left(X,t\right)}-\Psi_{0}>0$, then $w_{i}^{\left(2\right)}=1/n$, we have −2logℜ(Ψ_0_) = 0. When $\bar{\Psi_{1,A}\left(X,t\right)}-\Psi_{0}=0$, then −2logℜ(Ψ_0_) converges to $\chi_{1}^{2}$ in distribution. Otherwise, $\bar{\Psi_{1,A}\left(X,t\right)}$ converges to *N*(Ψ_1_, *σ*^2^) under *H*_0_.

1. When Ψ_1_ > Ψ_0_, $p(\bar{\Psi_{1,A}\left(X,t\right)}-\Psi_{0}\geq 0)\to 1.$
2. When Ψ_1_ = Ψ_0_, $p\left(\bar{\Psi_{1,A}\left(X,t\right)}-\Psi_{0}\geq 0\right)\to \frac{1}{2}.$

For any *c* > 0,

$$\begin{array}{ccc} & & \lim_{n\to \infty }p\left(-2\log ℜ\left(\Psi_{0}\right)\leq c\right)\\ & & \;=\lim_{n\to \infty }p\left(-2\log ℜ\left(\Psi_{0}\right)\leq c|\bar{\Psi_{1,A}\left(X,t\right)}-\Psi_{0}\geq 0\right)p\left(\bar{\Psi_{1,A}\left(X,t\right)}-\Psi_{0}\geq 0\right)\\ & & \;+\lim_{n\to \infty }p\left(-2\log ℜ\left(\Psi_{0}\right)\leq c|\bar{\Psi_{1,A}\left(X,t\right)}-\Psi_{0}<0\right)p\left(\bar{\Psi_{1,A}\left(X,t\right)}-\Psi_{0}<0\right)\\ & & \;=\frac{1}{2}+\frac{1}{2}p\left(\chi_{1}^{2}\leq c\right). \end{array}$$

The proof is finished.
